# Effect of surface and internal defects on the mechanical properties of metallic glasses

**DOI:** 10.1038/s41598-017-13410-3

**Published:** 2017-10-18

**Authors:** Sunghwan Kim, Seunghwa Ryu

**Affiliations:** Department of Mechanical Engineering, Korea Advanced Institute of Science and Technology (KAIST), 291 Daehak-ro, Yuseong-gu, Daejeon, 34141 Republic of Korea

## Abstract

Despite the significance of surface effects on the deformation behaviours of small-scale metallic glasses, systematic investigations on surface states are lacking. In this work, by employing atomistic simulations, we characterise the distributions of local inhomogeneity near surfaces created by casting and cutting, along with internal distributions in pristine and irradiated bulk specimens, and investigate the effects of inhomogeneity on the mechanical properties. The cast surface shows enhanced yield strength and degrees of shear localisation, while the cut surface shows the opposite effects, although the fraction of vibrational soft spots, known to indicate low-energy barriers for local rearrangement, is high near both surfaces. Correspondingly, plastic deformation is initiated near the cut surface, but far from the cast surface. We reveal that improved local orientational symmetry promotes strengthening in cast surfaces and originates from the effectively lower quenching rate due to faster diffusion near the surface. However, a significant correlation among vibrational soft spots, local symmetries, and the degree of shear localisation is found for the pristine and irradiated bulk materials. Our findings reveal the sensitivity of the surface state to the surface preparation methods, and indicate that particular care must be taken when studying metallic glasses containing free surfaces.

## Introduction

Metallic glasses are among the most promising candidates in material science for attaining high strength and high fracture toughness. Although metallic glasses exhibit these superior properties, the nature of sudden failure accompanied with shear bands and ductility tuning remain poorly understood^1–3^. Currently, macroscopic deformation along shear bands is considered to occur by the cooperative nucleation and growth of basic plasticity units, called shear transformation zones (STZs)^4,5^. It has been shown that the degree of strain localisation is strongly influenced by the distribution of short-range order (SRO) symmetry and the evolution of short-to-medium-range order^2,5,6^.

Deformation modes in metallic glasses, or the temporal and spatial dynamics of STZs, depend on extrinsic factors such as the quenching rate^7^, strain rate^8^, composition^2^, surface treatment^9^, and irradiation damage^10^, as revealed by experiments and computer simulations. Because of the difficulty of analysing atomic structures experimentally, many insights on metallic glasses have been gained by computer simulations. Although significant surface effects have been observed at small scales in both computational and experimental studies, most atomistic simulations consider metallic glasses in two- or three-dimensional (2 or 3D) specimens with full periodic boundary conditions (PBCs) because these ease the characterisation of distributions of structural and dynamical inhomogeneity^11,12^. In particular, the two different methods of casting and cutting used to creating side walls for simulated nanopillar geometries create significant changes in the degree of shear localisation^2,9^. Cast specimens show mechanical properties and shear localisations similar to those of pristine bulk metallic glasses, while those with cut surfaces show reduced strength and less localised deformation behaviours. Meanwhile, it has been shown that irradiation damage significantly affects the local SRO such that homogeneous flow becomes the favoured deformation mode^10^. Higher quenching rates and lower strain rates are also shown to promote homogeneous deformation^7,8^.

Various approaches have been used in atomistic simulations to quantify the structural origin of the changes in deformation modes of metallic glasses. The inherent heterogeneity of metallic glasses in full PBCs can be precisely categorised based on the concept of Kasper polyhedra^13^. Inherent structural symmetries, including the icosahedral order, are also parameterised by bond orientation analysis^14,15^. The participation ratio of the lowest vibrational modes is used to capture vibrational soft spots, which are fragile local clusters indicating potential STZ nucleation sites^16–18^. Irreversible plastic jumps are quantified by defining the atomic deformation gradient tensor^11,19^. Such plastic rearrangements accumulate until the local stress concentration field is strong enough for a cascade of STZs to evolve throughout the specimen. Recently, chemical heterogeneity has been used to explain the reaction barriers to the coalescence of STZs^20^. However, although surface effects have been shown to intensify as the specimen size is decreased in both computer simulations^21,22^ and experiments^23,24^, the spatial distribution of structural disorder near the specimen surface and its effect on the mechanical properties of metallic glass have not been investigated in detail.

Here, by employing atomistic simulations, we systematically study the distribution of various structural defects on the surface and within the bulk, and the effects of defect distribution on the mechanical properties, by examining metallic glasses prepared by four different methods. We consider the pristine bulk, the specimen with cut surfaces, the specimen with cast surfaces, and irradiated bulk, referred to as ‘pristine’, ‘cut’, ‘cast’, and ‘irradiated’ henceforth. For the clarification, we note that the ‘bulk’ in the present study represents nanoscale specimen with full PBCs. We carry out athermal mechanical simulations to characterise the inelastic transformation driven by external shear strain^11,25^, although realistic deformations in MGs are determined by the intricate coupling between thermal activation and external loading^8,12,26^. We find that cast surfaces show enhanced yield strength and degrees of shear localisation, while cut surfaces show the opposite effects, although the fractions of vibrational soft spots are increased near both surfaces. In addition, by analysing the spatial distribution of local stress fields, we find that the cascade of STZs, or shear localisation, is initiated near the surface for the cut at a smaller strain than in the pristine, while plastic deformation is initiated far from the surface in the cast. We reveal that the improved local orientation symmetry promotes the strengthening effect of the cast, and originates from the effectively lower quenching rate by faster diffusion near the surface. This implies that vibrational soft spots do not necessarily indicate potential STZ nucleation sites for specimens with free surfaces. On the contrary, a strong correlation among vibrational soft spots, local symmetries, and degrees of shear localisation is found for the pristine and irradiated. Our findings reveal the sensitivity of the surface state to the surface preparation method, and indicate that special care must be used when studying metallic glasses containing free surfaces.

## Results and Discussion

**Figure 1:**
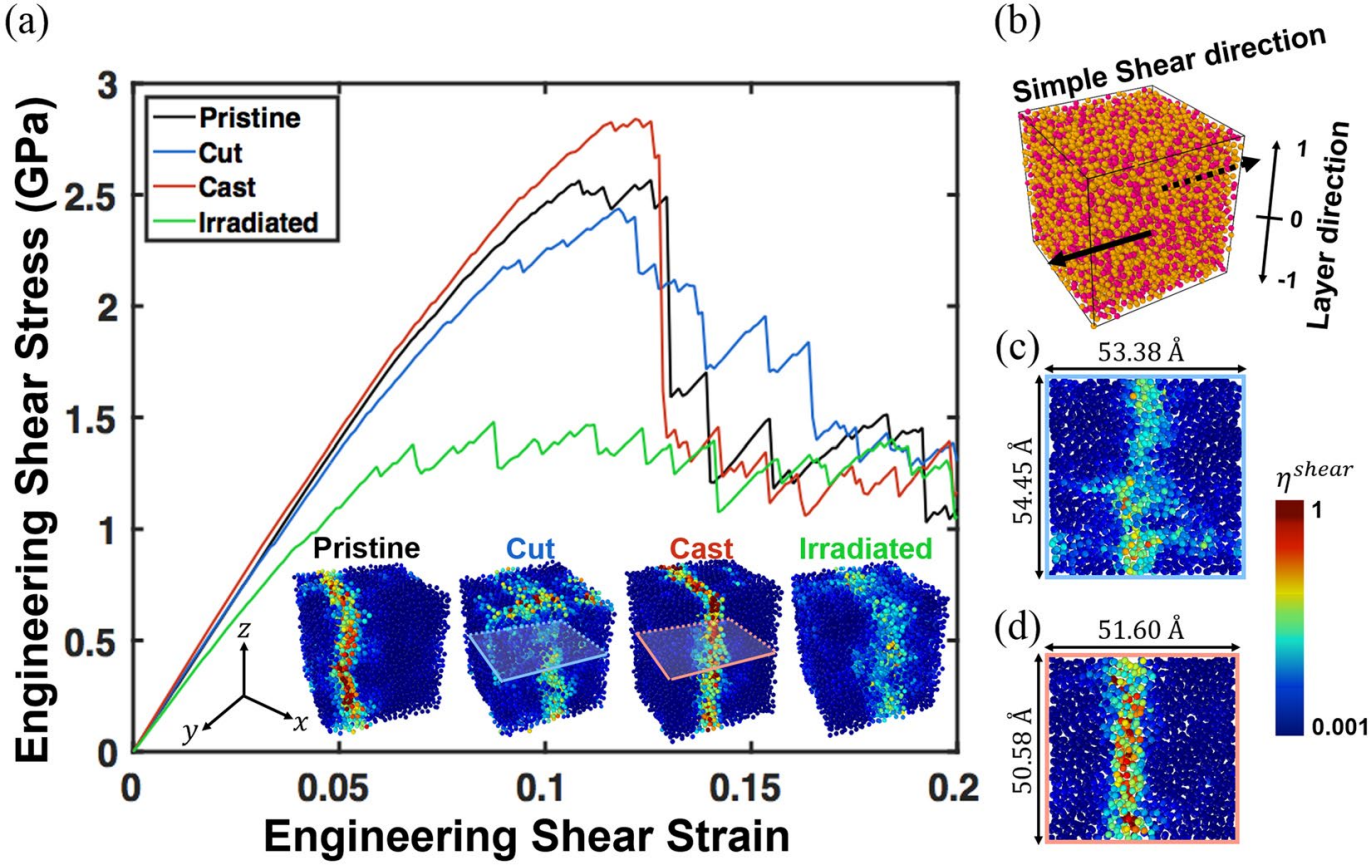
Shear stress–strain curve of each sample (**a**) Stress–strain curve by simple shear tests on the four different samples of pristine, cut, cast, and irradiated (left to right). Insets: Atomic shear strain distribution of each sample at 20% of engineering shear strain. (**b**) Schematic of simple shear tests and the layering division of the samples. The vertical axis is the *z*-axis, which is aperiodic for the cast and cut samples. Orange: Cu atoms; magenta: Zr atoms. Horizontal cross-sectional views of (**c**) cut and (**d**) cast samples at *z* = 0. For the visualisation purpose, the position of shear localisation is translated to the centre of the simulation box through periodic boundary condition in y-axis.

Figure 1a shows the stress–strain curves under simple shear tests of the four samples. The strength of the cut is smaller than that of the pristine, although the initial elastic responses are almost identical. The cut has a central microstructure identical to that of the pristine, but the surface region is expected to be softer because of the abrupt surface formation by the removal of the PBCs. The strength of the cast, however, is slightly larger than that of the pristine. The atoms near the surface have higher mobility, allowing more time to rearrange into lower-energy configurations, leading to a stiffening effect, as shown later. The irradiated shows significantly reduced stiffness and strength compared to the other samples, and exhibits more frequent activation steps (i.e. sudden discrete drops in stress) early in the shear test than the others. However, the flow stress at large strain is identical for all four specimen after the chains of STZs percolate the specimen, which is consistent with the literature^27^.

Previous studies have shown that the macroscopic strength is closely related to the order of the microscopic shear localisation^2^. Here, we represent the atomic strain tensor ${\eta }_{i}$ as ${\eta }_{i}=\frac{1}{2}({J}_{i}{J}_{i}^{T}-I)$, where ${J}_{i}$ denotes the local deformation gradient tensor^5^. The von Mises local shear invariant is obtained as^28^

1 $${\eta }^{shear}\equiv {\eta }_{i}^{Mises}=\sqrt{{\eta }_{yz}^{2}+{\eta }_{xz}^{2}+{\eta }_{xy}^{2}+\frac{{({\eta }_{yy}-{\eta }_{zz})}^{2}+{({\eta }_{xx}-{\eta }_{zz})}^{2}+{({\eta }_{xx}-{\eta }_{yy})}^{2}}{6}}$$

henceforth referred to as the atomic shear strain. The localised atomic shear strain of the samples at 20% applied shear strain (inset of Fig. 1a) shows that the cast exhibits a higher degree of shear localisation than the pristine does. However, the cut shows a much lower degree of shear localisation than the pristine, both centrally and near the free surface. This result implies the importance of an accurate consideration of the free-surface effects in predicting the deformation modes of metallic glasses. The surface states of the cut and cast significantly affect the degrees of shear localisation in the central planes, located ~27 $\AA$ away from the surfaces (Fig. 1c,d). In comparison, the irradiated has the least shear localisation. Our results show that the deformation mode of nanoscale metallic glass samples can be significantly affected by defects at both the exterior (surface treatment) and interior (irradiation processes) despite having identical chemical compositions and quenching rates.

**Figure 2:**
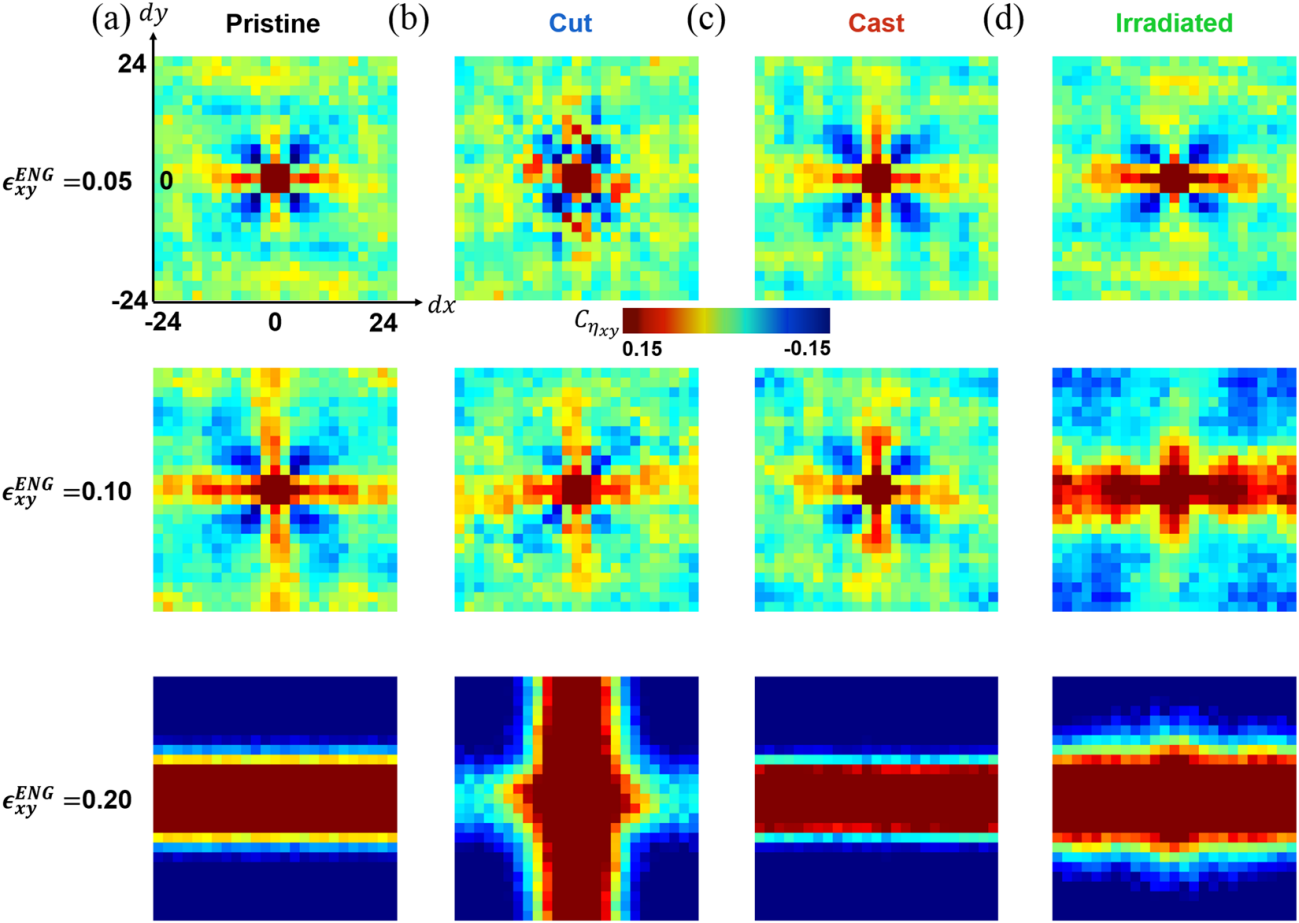
Illustration of spatial correlation of atomic shear strain of each sample. Illustrations of spatial correlation fields of atomic shear strain, ${{\boldsymbol{\eta }}}_{{\boldsymbol{xy}}}$, at (Top) 5%, (Middle) 10%, and (Bottom) 20% engineering shear strain of (**a**) pristine, (**b**) irradiated, (**c**) cut, and (**d**) cast.

For a more detailed analysis of the plastic deformation, we investigate the spatial correlation function of atomic strain for all samples, as depicted in Fig. 2. The shear stress around local isolated inclusions with different moduli has four-fold symmetry. For example, the stress distribution of an elastic medium with a circular hole (i.e. void inclusion) with the radius ${\rm{R}}$ under the pure shear stress S is given as

2 $${\sigma }_{xy}=S(1-\frac{2{R}^{2}}{{r}^{2}}+\frac{3{R}^{4}}{{r}^{4}}){\sin }^{2}2\theta +S(1+\frac{2{R}^{2}}{{r}^{2}}-\frac{3{R}^{4}}{{r}^{4}}){\cos }^{2}2\theta ,\quad {\rm{when}}\,r > R$$

where ${\rm{r}}=\sqrt{{x}^{2}+{y}^{2}}$ is the distance from the centre of the inclusion, and ${\rm{\theta }}={\tan }^{-1}\frac{y}{x}$ ^29^. Hence, in the presence of multiple isolated inclusions considered as STZs, the spatial correlation for the atomic strain has four-fold symmetry. The spatial correlation function for the atomic strain tensor component ${\eta }_{xy}$ is defined as

3 $${C}_{A}(d\vec{r})=\frac{\langle A(\vec{r}+d\vec{r})A(\vec{r})\rangle -{\langle A(\vec{r})\rangle }^{2}}{\langle A{(\vec{r})}^{2}\rangle -{\langle A(\vec{r})\rangle }^{2}}$$

where ${\rm{A}}={\eta }_{xy}$ and represent the averages over all atoms in the simulation cell^30^. The formation of STZs can also be visualised using the correlation function for the non-affine displacement ${D}_{min}^{2}$ (see Supplementary Information). Figure 2a–d represent the spatial correlation of ${\eta }_{xy}$ for the pristine, cut, cast, and irradiated obtained for $d\overrightarrow{r}=(dx,dy,0)$. From top to bottom, the correlation fields are shown at 5%, 10%, and 20% engineering shear strain. The correlation functions for the pristine and cast maintain clear four-fold symmetry up to 10% strain, as depicted in Fig. 2a and c, while the correlation fields of the non-affine displacement ${D}_{min}^{2}$ are isotropic, as shown in Supplementary Figs 1–8. The four-fold symmetry is abruptly broken when the STZ cascade is initiated, and the correlation functions form a narrow band at 20% strain. On the contrary, as shown in Fig. 2b and d, the cut and irradiated have distorted four-fold symmetries along the $dx$ direction at 5% strain, indicating the occurrence of some STZ cascades at this low strain. Interestingly, the shear localisation proceeds along the same axis for the irradiated, while the orientation of localisation takes the direction of the $dy$-axis in the cut. Such behaviour can be directly visualised from the apparently shear-transformed atoms (atoms with large plastic jumps, as defined formally later) in the Supplementary Figs 3–4. The STZ cascade typically proceeds in a consistent direction once initiated, because the stress concentration around an ellipsoidal inclusion (such as an STZ chain) is highest near the most prolonged tip of the inclusion. However, the cut sample has two distinct regions of the stiff centre and soft surface. While the initial plastic deformation and shear localisation occur near the surface at low applied stress, the STZ cascade cannot be maintained because the required stress for STZ formation is significantly higher in the central region. A new STZ cascade is nucleated throughout the central region at a higher stress; this may proceed along a different direction. The detailed evolution of the correlation function and the apparently shear-transformed atoms can be found in Supplementary Figs 1–8.

**Figure 3:**
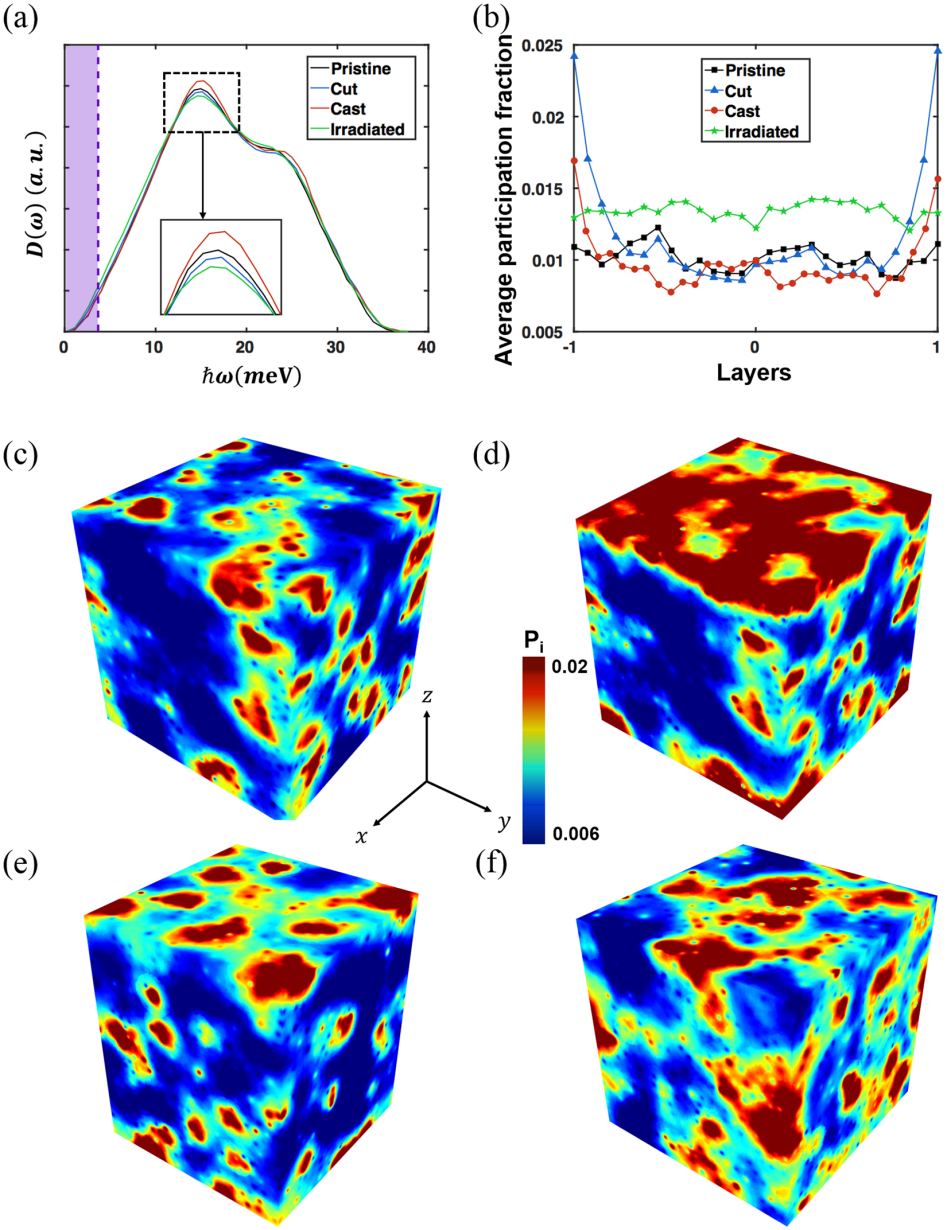
Vibrational densities of states and resulting participation fraction distribution. (**a**) V-DOS of pristine, cut, cast, and irradiated. It is noted that the eigenmodes corresponding to the 1% lowest eigenvalues are selected to calculate the participation ratio. The eigenmodes participating in the purple region are used to calculate the participation fraction. (**b**) Averaged participation fraction along the layers of each sample. 3D graphical representation of the distribution of the participation fraction for (**c**) pristine, (**d**) cut, (**e**) cast, and (**f**) irradiated.

In order to investigate the structural origin of the observed plastic deformation, we examine the local stiffness values of the samples based on the vibrational density of states (V-DOS), as represented in Fig. 3. Sharper first peaks in the V-DOS generally correlate to the involvement of fewer low-frequency modes in the sample^17^. As expected from the strengths of the four samples, the first peak of the cast is the sharpest, as shown in Fig. 3a. However, the internal structural defects in the irradiated blunt the first peak, corresponding to a significant increase in the fraction of low-frequency modes. The increasing order of defects seems to have effects similar to those of the increasing quenching rate reported in the literature^17^. The participation fraction ${P}_{\lambda }$ is defined as ${P}_{\lambda }=\frac{1}{n}{[\sum _{i\in {\rm{\Omega }}}({\vec{e}}_{i}(\lambda )\cdot {\vec{e}}_{i}(\lambda ))]}^{-1}$, where ${\rm{\lambda }}$ defines the normal mode of the atoms (${\rm{\lambda }}$ = ~1–3*N*_*tot*_, where *N*_*tot*_ denotes the total number of atoms), ${\overrightarrow{e}}_{i}(\lambda )$ represents the eigenvectors from the Hessian matrix constructed from atomic perturbation ($i$ = ~1–3*N*_*tot*_), ${\rm{\Omega }}$ is the set of selected elements whose vibration energy $\hslash w$ is smaller than the lowest 1% value of 3.71 meV in the pristine, and $n$ is the cardinality of the set ${\rm{\Omega }}$ ^18^. We use the same cutoff value of $\hslash w$ for the four samples to accommodate the relative differences in vibrational soft spots among the samples. In order to consider the spatial distribution of vibrational soft spots along the aperiodic axes, we subdivide the samples into layers orthogonal to the surface, as depicted in Fig. 1b. The central plane of the layer direction (*z*-axis) is set to 0 and the distances to each edge are normalised to $\pm 1$. Figure 3b presents the variation of the participation fraction for the 1% lowest vibrational frequency modes through the layers. We find that the averaged participation ratio rises near the surface in for both the cast and cut. In addition, the cut shows a higher participation ratio near the surface than the cast does, originating from the abrupt surface creation. The pristine and irradiated show homogeneous distributions of soft spots, as expected. In the absence of a free surface, we can state that, because of the larger fraction of vibrational soft spots, STZ nucleation occurs more uniformly in the irradiated than in the pristine, eventually reducing the degree of shear localisation. However, although the vibrational soft spot fraction is higher at the casted surface, plastic deformation is not initiated near the surface and the degree of shear localisation is higher than that in the pristine. This implies that the vibrational soft spots do not necessarily indicate potential STZ nucleation sites for specimens with free surfaces. 3D views of the participation fraction distributions of the samples are illustrated in Fig. 3c–f.

**Figure 4:**
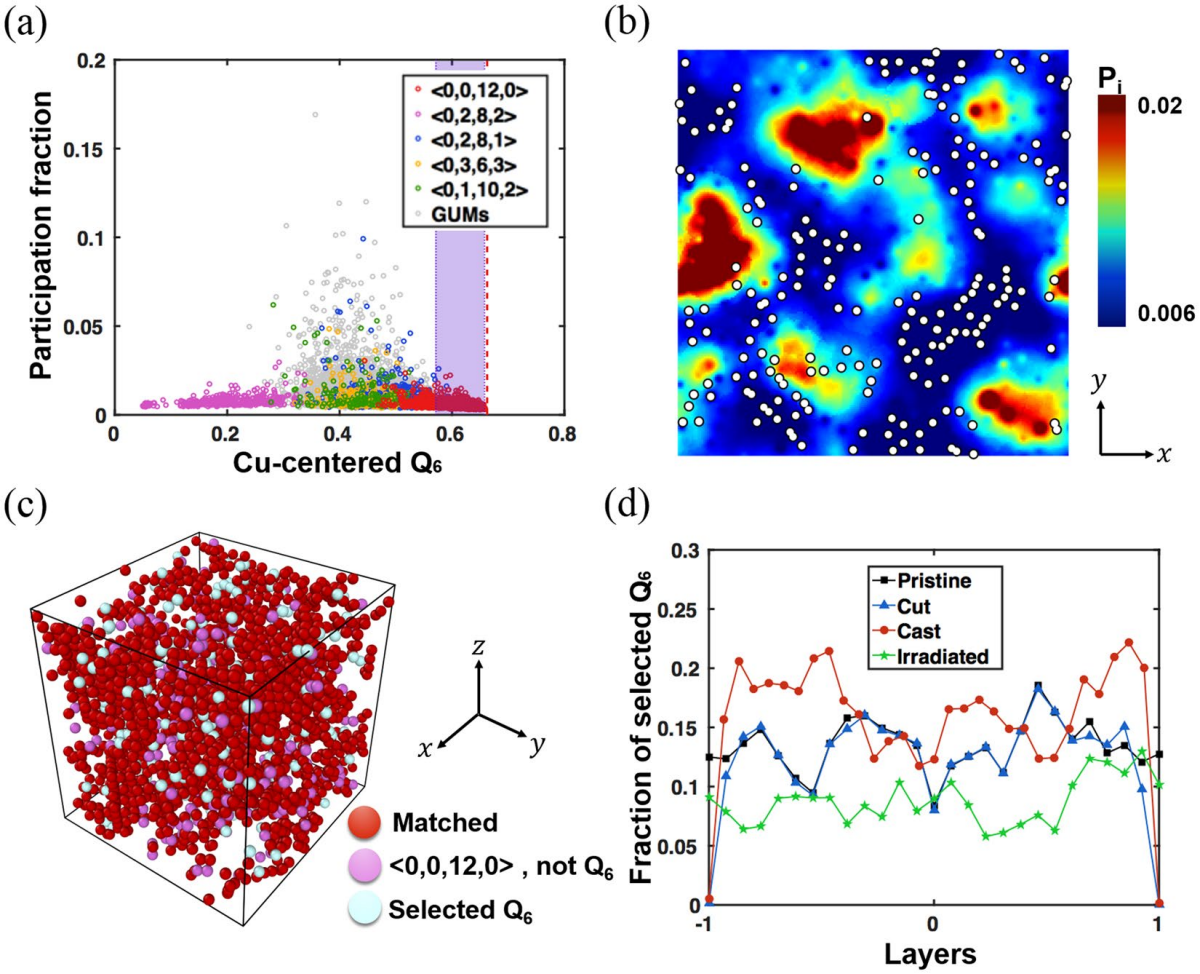
Schematic defining Q_6_ and relationship thereof to typical locally favoured motifs. (**a**) Distribution of the participation fraction along the Cu-centred Q_6_. Different colouring represents the top five most-favoured Cu-centred motifs including perfect icosahedra, <0, 0, 12, 0>. The selected range with the purple box, [0.57174, 0.65836], is chosen to have the same amount of atoms as the total number of Cu-centred icosahedra in the pristine sample. The red dotted line at Q_6_ = 0.663 represents the ideal icosahedron. (**b**) An illustration of the atoms with the selected Q_6_ and the distribution of participation fraction. The white circles show the atoms in the selected Q_6_ range. Thin slabs of the pristine bulk sample have thicknesses of 5 $\AA$. (**c**) Schematic of the atoms belonging to the selected Cu-centred Q_6_ and the Cu-centred icosahedra in the pristine. Red colouring indicates that atoms belong to both Q_6_ and Cu-centred icosahedra. (**d**) Fraction of the selected Cu-centred Q_6_ parameters along the layers of each sample.

Next, we examined the spatial distribution of local orientation symmetry to investigate the origins of different mechanical properties in the four specimens. The Voronoi tessellation, a typical method for determining the Kasper polyhedra, cannot be clearly defined for aperiodic boundaries, because of ambiguities in defining the surface encompassing the total volume. Instead, we adopted the BOO parameter Q_6_, which can be defined only with neighbouring atomic arrangements^14^. We first examined the correlation between the Cu-centred Q_6_ and various Cu-centred locally favoured motifs as well as geometrically unfavoured motifs (GUMs) known for the Cu_64_Zr_36_ system for the pristine (Fig. 4a)^6^. Because the Cu-centred icosahedron is known as the most stable local cluster in the selected chemical composition, we attempted to capture the local structures from the BOO parameter Q_6_ = 0.663, corresponding to perfect icosahedral symmetry^15^. We define the range of BOO parameters to include slightly distorted Cu-centred icosahedra and test the correlation between the icosahedral order found from both Voronoi tessellation and the Q_6_ parameter, as shown in Fig. 4a–c and Supplementary Fig. 9a,b. The range of Cu-centred Q_6_ is determined to include the same amount of atoms as the total number of Cu-centred full icosahedra from the Voronoi tessellation in the pristine. In the range considered (${{\rm{Q}}}_{6}\, > \,0.57174$), the atoms selected by Cu-centred icosahedra from Voronoi tessellation and the atoms in the selected Q_6_ range match at 85% of sites, as shown in Fig. 4c. We also find that Cu atoms with higher Q_6_ parameters, illustrated by white circles, are located at the low-participation-fraction sites, i.e. sites with higher local structural rigidity, in the pristine, as shown in Fig. 4b and in all samples in Supplementary Fig. 10. The correlation between Zr-centred Q_6_ and the participation fraction is depicted in Supplementary Fig. 9a, where no strong correlation is seen. Having established the validity of using the Q_6_ parameter to identify local icosahedral order, we calculate the fraction of selected Q_6_ parameters found along the layer of the specimen containing free surfaces (Fig. 4d). We find that more stable microstructures, i.e. higher local symmetries, are developed near the cast surface, except in the outermost layers. In the cast, the favourable SROs developed near the surface reduce the surface nucleation of STZs, as shown in Supplementary Figs 5 and 6.

**Figure 5:**
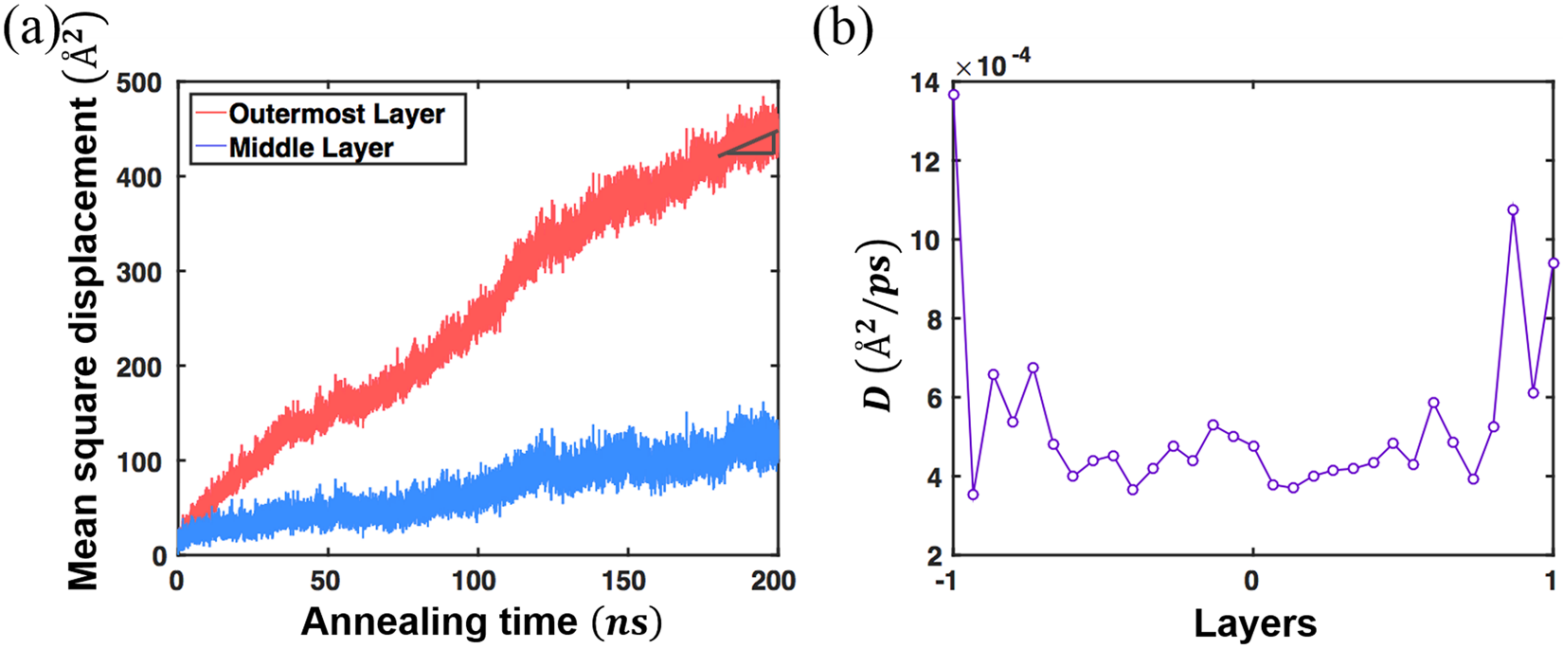
Mean square displacement and resulting self-diffusivity of the cast at 700 K. (**a**) MSD of all atoms in the cast as a function of the annealing time. (**b**) Self-diffusivity along the layers of the cast.

In order to understand the origin of enhanced symmetry near the cast surface, we calculated the self-diffusivity constant *D* for the cast at 700 K, below the glass transition temperature (~745 K) of the selected chemical composition^2^. The self-diffusivity *D* is defined as

4 $$D=\mathop{\mathrm{lim}}\limits_{}\frac{\langle {|\vec{r}(t+{t}_{0})-\vec{r}(t)|}^{2}\rangle }{6t}$$

where the numerator is the definition of the mean square displacement (MSD), 〈 〉 symbolises the average over all atoms in the cast, and the initial time is *t*_0_^31^. Figure 5a presents the MSDs during the 200 ns of NVT simulations at 700 K obtained at the outermost and central layers of the cast. The atoms near the surface travel significantly higher diffusion distances than those near the central region. *D* along the aperiodic layer is then calculated, using the last 20 ns of the simulation time, as shown in Fig. 5b. Interestingly, *D* in the outermost layer is ~5 times larger than that in the central layer. This implies that the atoms near the surface remain mobile even below the glass transition temperature. Hence, the atoms near the surface experience much longer effective structural relaxation times, and form higher-symmetry structures after the quenching process. We note that, when the cut sample is annealed at 700 K for 200 ns, structural properties and mechanical responses become almost identical to the cast sample, which also proves that the facile atomic diffusion at surfaces is responsible for the higher structural symmetry and stiffer mechanical response of the cast surface as shown in Supplementary Fig. 11. Because such relaxation process occurs much slower at 300 K, we observe significantly less changes on structural and mechanical properties after 300 K annealing.

**Figure 6:**
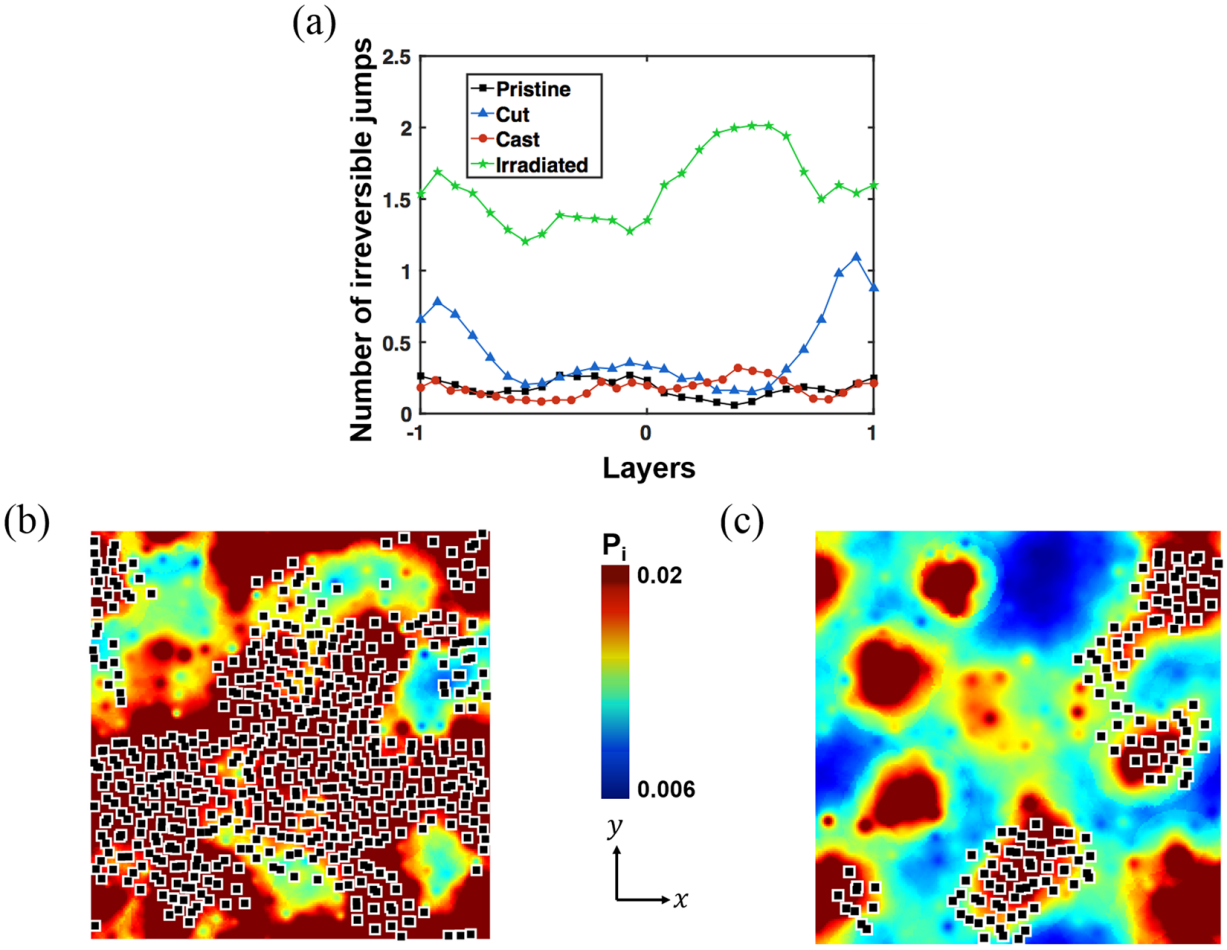
Distribution of non-affine displacements and resulting irreversible (plastic) jumps during the simple shear tests. (**a**) Averaged number of irreversible jumps along the layers of each sample. Illustrations of the irreversible jumps and the distribution of participation fractions on the surface layer of (**b**) the cut and (**c**) cast. Black cubes indicate atoms experiencing irreversible jumps. Thin slabs of the cut and cast samples have the thickness of 5 $\AA$.

Figure 6 presents the correlation between the event of irreversible atomic rearrangement and the soft spots, based on the participation fraction analysis. Supplementary Fig. 12a represents the frequency of the non-affine displacement accumulated for up to 10% engineering shear strain. The non-affine displacement is defined from ${J}_{i}$ as

5 $${D}_{\min \,,i}^{2}=\frac{1}{{N}_{i}}{[{\vec{r}}_{j}(t)-{\vec{r}}_{i}(t)-{J}_{i}({\vec{r}}_{j}(t-{\rm{\Delta }}t)-{\vec{r}}_{i}(t-{\rm{\Delta }}t))]}^{2}$$

where ${N}_{i}$ is the number of nearest neighbours^5^ and the reference configuration is taken from the previous strain step. We define the threshold of non-affine displacement at the value of 0.1 ${{\rm{\AA }}}^{2}$, shown as a dotted purple line, as indicated in Supplementary Fig. 12. Figure 6a represents the distribution of the number of plastic events along the aperiodic axis. In the cut, most highly vibrational soft spots, coloured red, undergo the plastic event, while a smaller fraction of highly vibrational soft spots does so in the cast. While the plastic events are first localised near the surfaces before proceeding to the central region for the cut, the cast exhibits dominant plastic events far from the surface because of the lower structural symmetry (lower BOO) in the central region (see Supplementary Figs 3–6 for details). As depicted in Fig. 6b, the cut exhibits frequent surface-driven plastic rearrangement early in strain application. The irradiated, on the other hand, exhibits uniform plastic jump distribution along all layers. The time evolutions of the plastic jump distributions for the four samples are illustrated in Supplementary Fig. 13. We note that the width of the apparently shear-transformed atoms near the cut surface remains almost the same at ~15 $\AA$, until the STZ cascade completely percolates the central region. In other words, the softening from the abrupt surface formation extends ~15 $\AA$ from the surface. Hence, for metallic glasses in atomistic simulation with sizes comparable to the softening depth, the degree of shear localisation would be affected significantly.

We expect a similar effect by surface softening for the metallic glass samples tested in experiments. For nanopillars experimentally prepared by the focused ion beam (FIB) method, the depth of softening would be significantly higher, which explains the enhanced plasticity of FIB sample at 100 nm scale^32^. In contrast, 300 nm or larger diameter structures do not show significant changes in deformation behaviour under compression or tension regardless of FIB damage, suggesting that the FIB damage affects the local symmetry in the region much shallower than 300 nm^23,32,33^. In contrast, significant plasticity at 400 nm scale was achieved when the interior of the specimen is also damaged via a series of proton irradiation processes^10^. Our annealing simulations on the cut sample (Supplementary Fig. 11) imply that the surface damage by FIB may be recovered after long time relaxation process at high temperature, and the critical size for the intermittent-to-homogeneous deformation^23,33^ can be increased.

## Summary and Conclusion

We have investigated defect distributions and the effects thereof on the mechanical properties for pristine, cut, cast, and irradiated Cu_64_Zr_36_ metallic glasses. We find that vibrational soft spots are concentrated near the surfaces, regardless of the preparation methods, because of broken bonds at the surfaces. Meanwhile, local orientational order is enhanced only near the cast surface, because of the longer effective relaxation time originating from the higher atomic mobility near the surface. Hence, the rigidity and the degree of shear localisation are enhanced for the cast compared to the pristine, while they are diminished for the cut. This implies that vibrational soft spots do not necessarily indicate low orientational order or preferential STZ nucleation sites in the presence of free surfaces. The irradiated experiences homogeneous damage throughout the volume; the reduced rigidity and lower degree of shear localisation can be understood from the correlation among vibrational soft spots, low orientational order, and preferential STZ nucleation sites. Our findings imply that metallic glass specimens with sizes comparable to the surface damage depth (15 $\AA$ for the cut sample in the present study) experience lower degrees of shear localisation and higher ductility. In real experiments where surface damage from FIB or other cutting methods is deeper than that in the present study, abrupt changes in the deformation modes, such as brittle-to-ductile transitions, may occur at larger scales. In the future work, by employing the advanced sampling techniques such as autonomous basin climbing (ABC) methods for accessing experimental strain rate, we plan to investigate the rate-dependent intermittent-to-homogeneous deformation transition observed in experiments^8,34^.

## Methods

We studied the Cu_64_Zr_36_ metallic glass because it has a relatively high density of five-fold locally favoured motifs called icosahedra^2^. The Large-scale Atomic/Molecular Massively Parallel Simulator (LAMMPS) was employed to perform molecular statics/dynamics simulations^35^. The embedded-atom-method (EAM) interatomic potential, developed by Sheng *et al*., was selected to describe interatomic interactions in the CuZr binary system^2^. In order to successfully examine the surface effects, we used high surface-area-to-volume-ratio cubic specimens with dimensions of ~54 $\AA$ × 54 $\AA$ × 54 $\AA$ containing 10,000 atoms. Each sample in the study was equilibrated for 10 ns at 2000 K and quenched to 300 K at the rate of 10^9^ K/s. We chose the quenching rate of 10^9^ K/s as a computationally limiting case to have well-relaxed glass structure^36^. All stress components along the periodic boundaries were relaxed during the quenching processes.

The four types of metallic glass samples of the pristine, cut, cast, and irradiated are depicted in Fig. 1. Four samples represent the pristine, with PBCs along all three axes; two samples (cut and cast) have free boundaries along the *z*-axis and PBCs along the *x*- and *y*-axes; another pristine bulk sample incorporates irradiation damage. The cut is prepared by abruptly turning off the PBC along the *z*-axis from the pristine after quenching. The cast is produced by cutting the sample immediately after the 2000 K equilibration process and quenching it in the presence of the external harmonic spring potential wall represented by k(z−r_c_)^2^ along the aperiodic direction where ${\rm{z}}$ is the distance from the central *x*-*y* plane of the specimen. The potential wall is activated when ${\rm{z}}$ is larger than the cutoff distance *r*_*c*_. The effective spring constant ${\rm{k}}$ is set to $0.5\,{\rm{eV}}/{\AA }^{2}$ and *r*_*c*_ is defined as one-half the vertical length of the sample at ~28 $\AA$ to confine the atoms during the quenching period. The irradiated is constructed by applying a series of atomic bombardments to the pristine bulk. For a bombardment event, a velocity corresponding to 100 eV in energy is assigned to a randomly chosen atom in a random direction. Then, the sample is equilibrated for 1.5 ps with the microcanonical ensemble (NVE) and 3.0 ps with the isothermal–isobaric ensemble (NPT) sequentially. This bombardment process is repeated 200 times to reproduce a certain level of structural transitions^10^. The short-range pairwise Lennard–Jones potential (${{\rm{\varepsilon }}}_{{\rm{LJ}}}=2.0$ $\AA ,{{\rm{\sigma }}}_{{\rm{LJ}}}=1.871$ eV) is augmented during the irradiation process to resolve the soft core problem of the EAM potential. The quenched samples at 300 K are then relaxed using a conjugate gradient. Athermal quasi-static simple shear tests (AQS) are performed in the *x-y* plane by gradually changing the shape of the simulation cell with incremental steps of 0.001 of the engineering strain (${\epsilon }_{{\rm{xy}}}^{ENG}$).

The structural order was characterised by various measures. We obtained the entire normal mode spectrum from the full Hessian matrix and identified soft vibrational modes from the participation ratio of each atom at the vibration energy corresponding to the lowest 1% normal-mode frequency of the pristine^17^. With Voronoi tessellation, we obtained the Kasper polyhedron for each atom, from which we could identify local icosahedral structures in the pristine. Because of the ambiguity of defining boundaries by Voronoi tessellation, the local icosahedral order for the cut and cast were analysed by the Q_6_ parameter from the bond orientational order (BOO) analysis^14,15^, after establishing its correlation with the Kasper polyhedron in the pristine. The local deformation gradient tensor ${J}_{i}$ was used to define the atomic strain tensor ${\eta }_{i}$ and the non-affine displacement ${D}_{min}^{2}$ ^5^. ${J}_{i}$ mapped the relative displacement against the reference, i.e. ${J}_{i}:\{{d}_{ji}^{t+{\rm{\Delta }}t}\}\to \{{d}_{ji}^{t}\}$, $\forall j\in {N}_{i}$, where ${N}_{i}$ is the number of nearest neighbours of the *i* ^th^ atom. The cutoff distance for assigning ${N}_{i}$ was chosen as the first minimum position of the radial distribution function of the pristine bulk sample of 3.5 $\AA$. In this study, the reference state for the atomic strain tensor was fixed at the initial zero-strain state, while the reference for non-affine displacement was the configuration at the previous strain step. We defined the apparently shear-transformed atoms as those with accumulated ${D}_{min}^{2}$ values over the threshold of 0.1 ${\AA }^{2}$ until reaching 10% of the applied (engineering) shear strain. These atoms were likely within STZs. The total number of irreversible jumps was captured by counting the number of apparently shear-transformed atoms^37^.

## Electronic supplementary material

Supplementary Information
