# Supplementary material for: Effect of surface and internal defects on the mechanical properties of metallic glasses
Source: Sci Rep. 2017 Oct 18;7:13472. doi: 10.1038/s41598-017-13410-3 (PMC5647394; doi:10.1038/s41598-017-13410-3)
Supplement: Supplementary file 1 — Supplementary Information [file 41598_2017_13410_MOESM1_ESM.pdf]

Supplementary Information

Effect of surface and internal defects on the mechanical properties of metallic glasses

Sunghwan Kim[1] and Seunghwa Ryu[*,1]

Affiliations

[1] Department of Mechanical Engineering, Korea Advanced Institute of Science and Technology (KAIST), 291 Daehak-ro, Yuseong-gu, Daejeon 34141, Republic of Korea

[*]Corresponding author email : ryush@kaist.ac.kr

Supplementary Figure 1: The selected engineering strain for the correlation analysis in the pristine

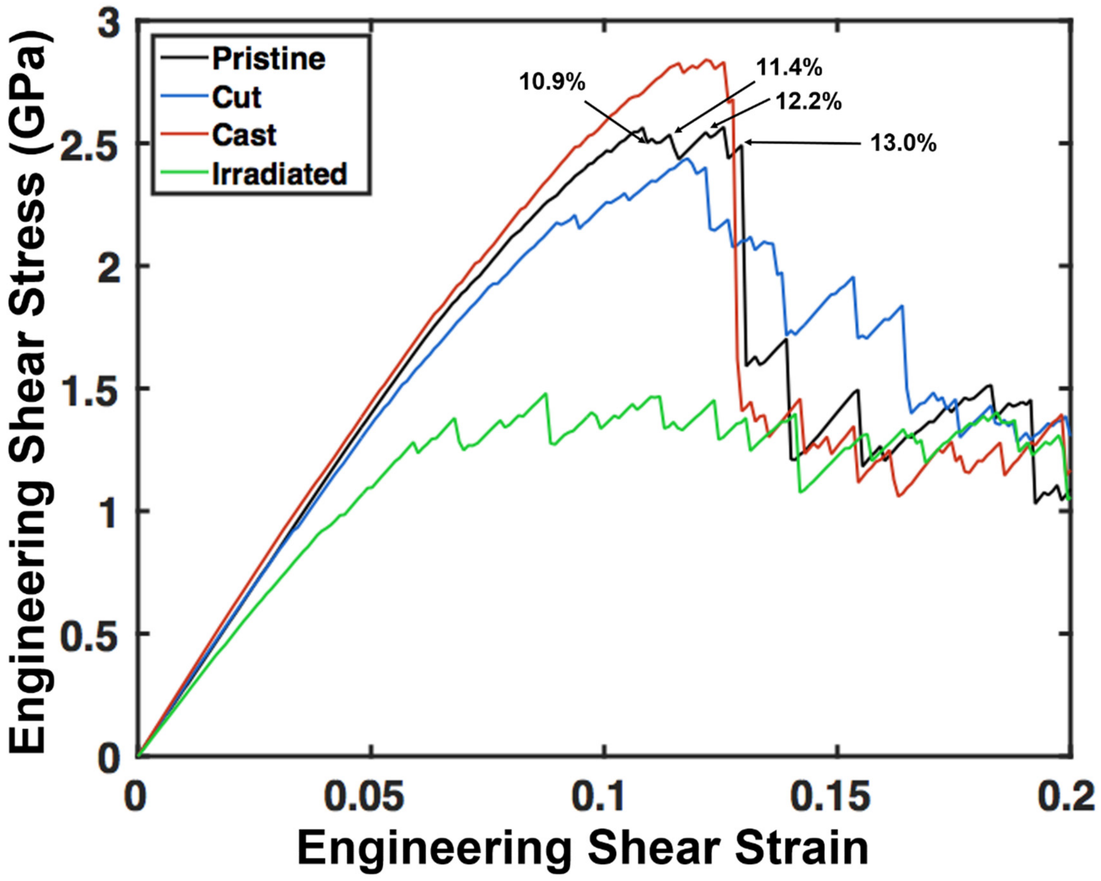

Supplementary Figure S1. 0.2% (Elastic range), 10.9%, 11.4%, 12.2%, 13.0% engineering shear strain steps are selected to analyse the correlation fields of the pristine.

Supplementary Figure 2: The correlation fields of atomic shear strain and non-affine displacements and corresponding configurational state in the pristine.

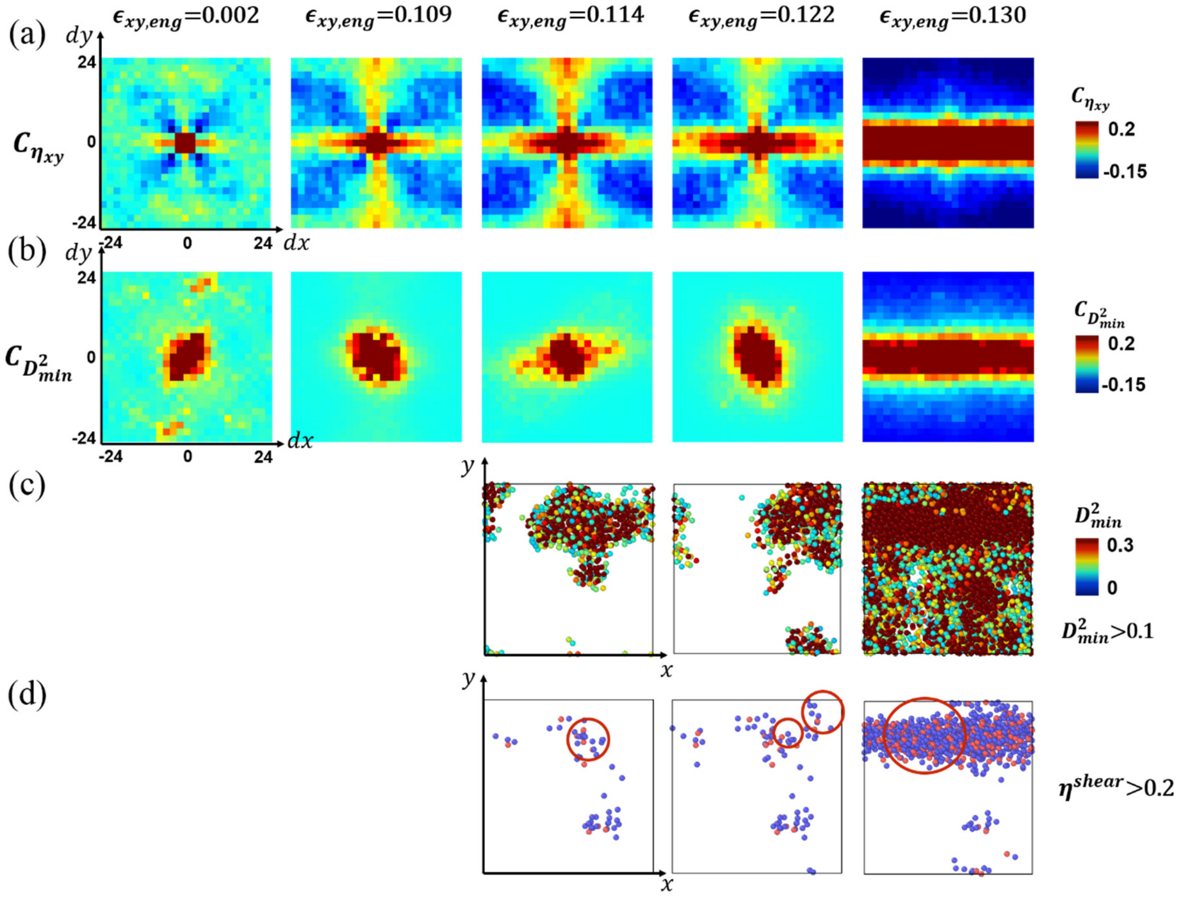

Supplementary Figure S2. The correlation fields of (a) atomic shear strain and (b) non-affine displacements in the pristine. The views in xy-plane with the criterion of (c) non-affine displacements and (d) the atomic shear strain. Note that the marked red circles represent the activated atoms from the previous engineering strain step.

Supplementary Figure 3: The selected engineering strain for the correlation analysis in the cut

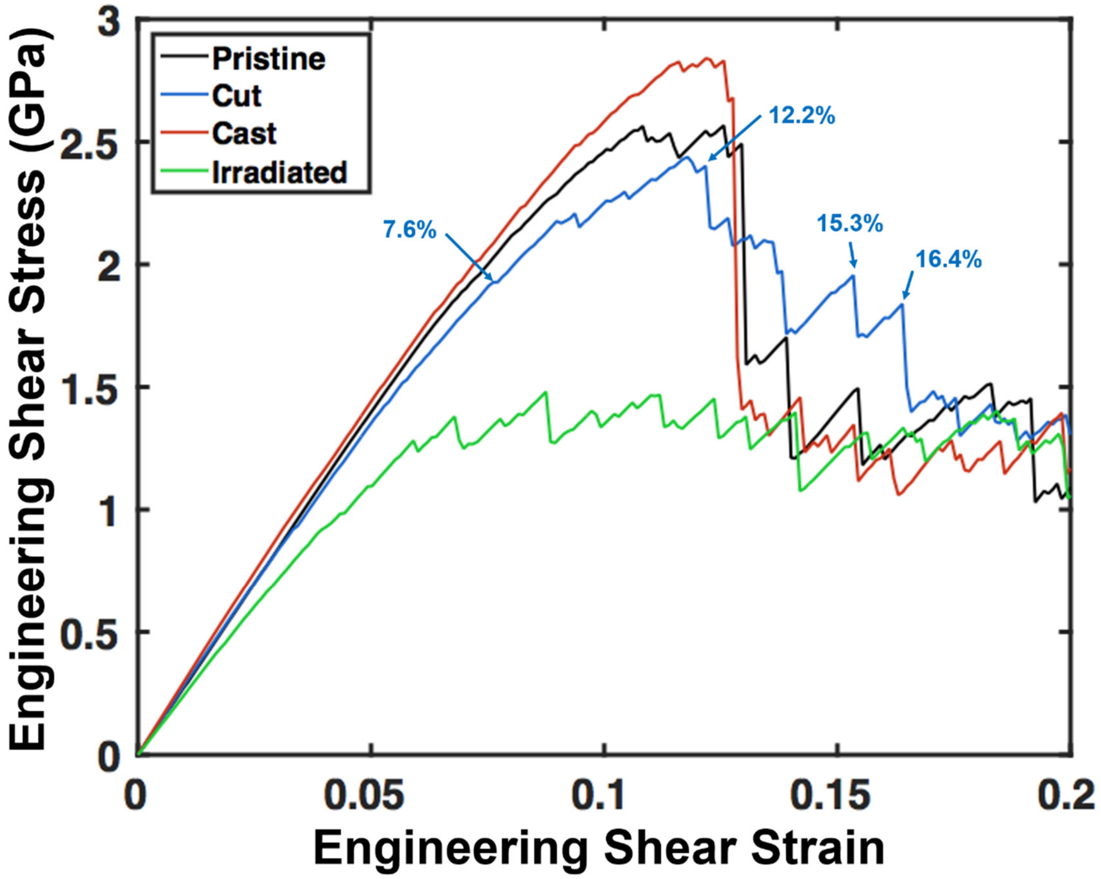

Supplementary Figure S3. 0.2% (Elastic range), 7.6%, 12.2%, 15.3%, 16.4% engineering shear strain steps are selected to analyse the correlation fields of the cut.

Supplementary Figure 4: The correlation fields of atomic shear strain and non-affine displacements and corresponding configurational state in the cut.

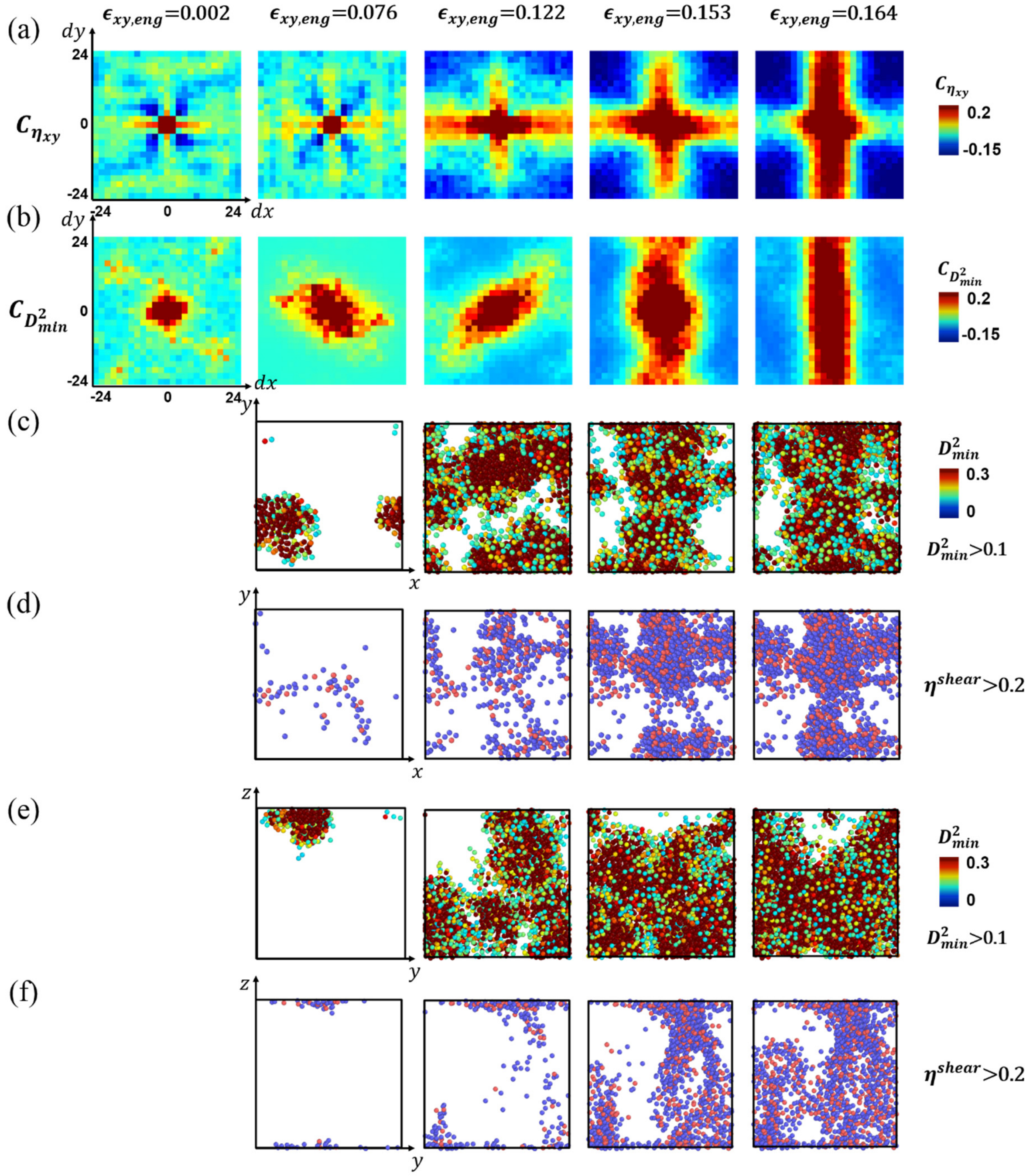

Supplementary Figure S4. The correlation fields of (a) atomic shear strain and (b) non-affine displacements in the cut. The views in xy-plane with the criterion of (c) non-affine displacements and (d) the atomic shear strain. The views in yz-plane of (e) the non-affine displacements and (f) the atomic shear strain illustration following. Note that the configurational states illustrated in (e) and (f) are in yz-plane to show the surface state.

Supplementary Figure 5: The selected engineering strain for the correlation analysis in the cast

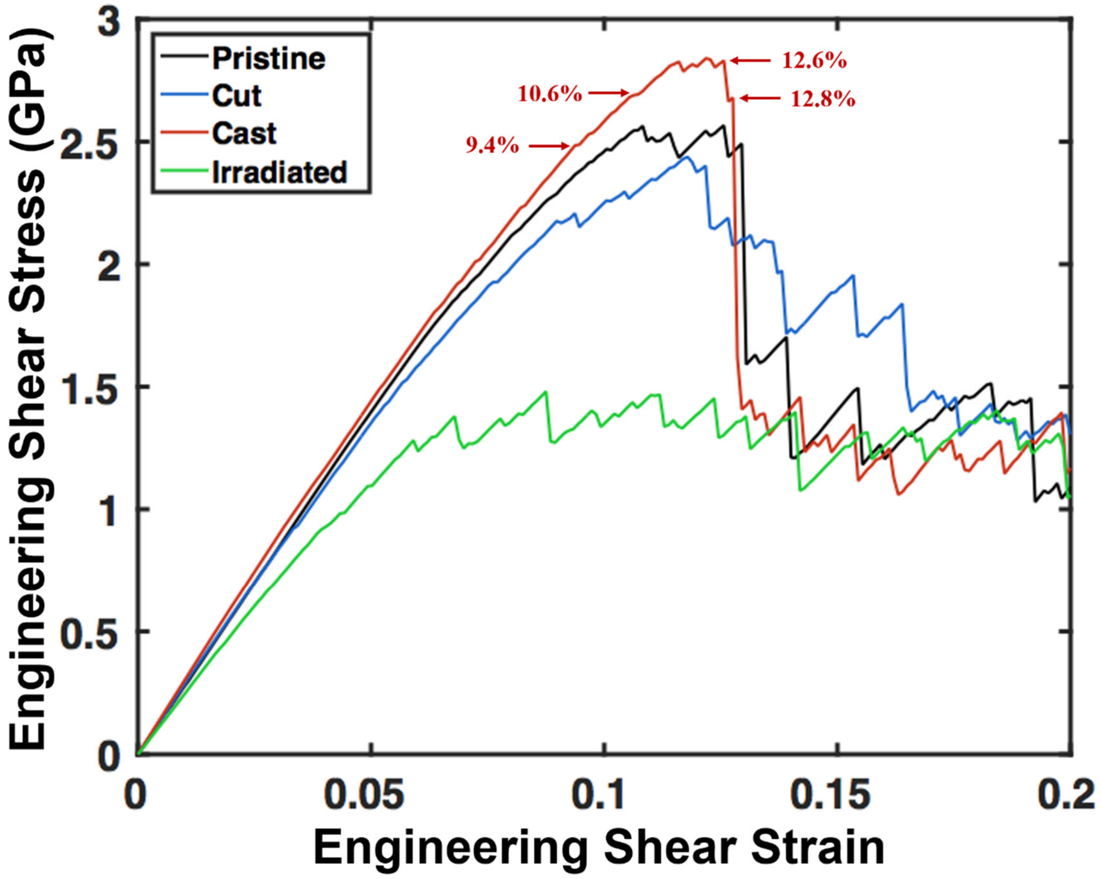

Supplementary Figure S5. 0.2% (Elastic range), 9.4%, 10.6%, 12.6%, 12.8% engineering shear strain steps are selected to analyse the correlation fields of the cast.

Supplementary Figure 6: The correlation fields of atomic shear strain and non-affine displacements and corresponding configurational state in the cast.

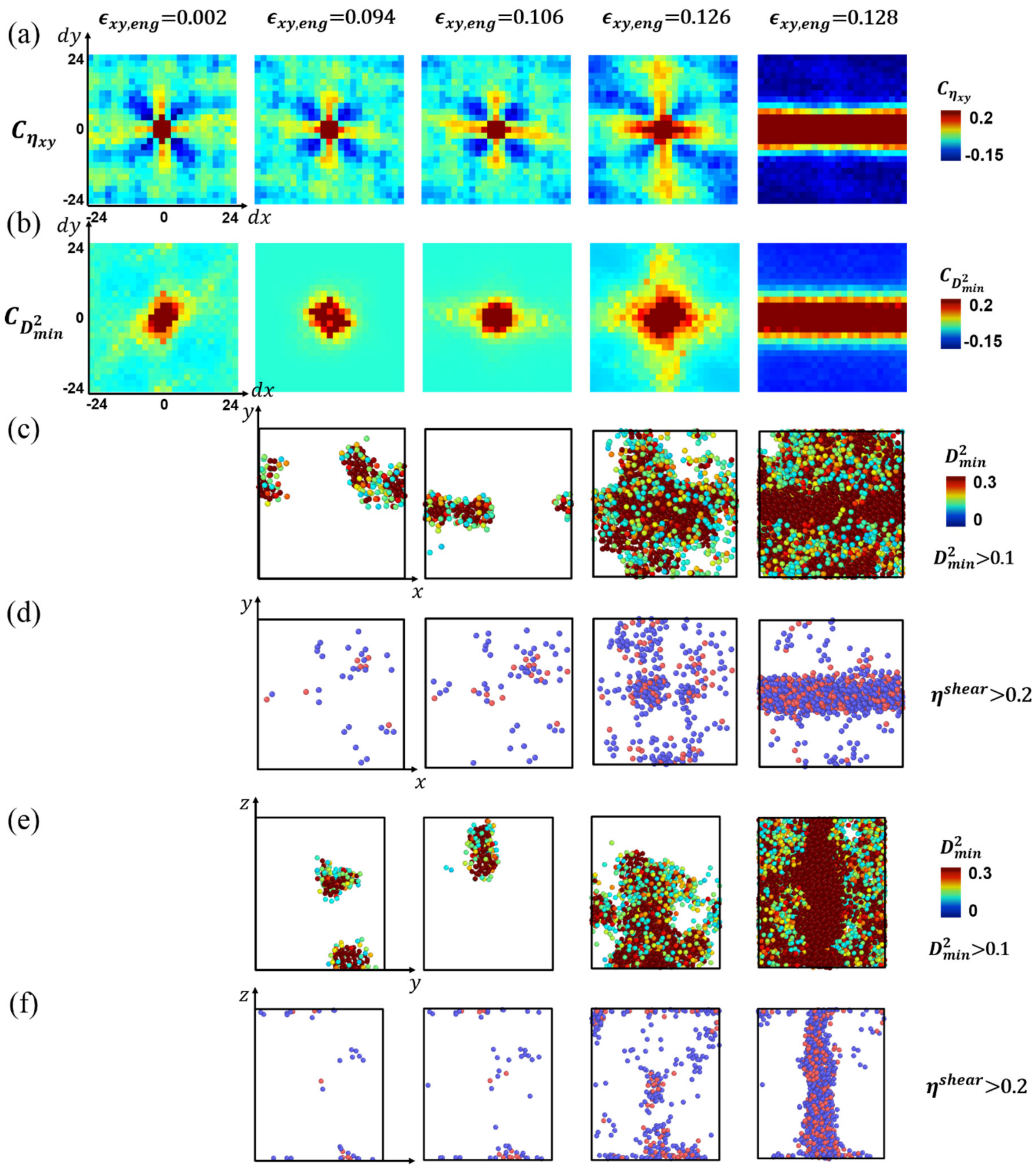

Supplementary Figure S6. The correlation fields of (a) atomic shear strain and (b) non-affine displacements in the cast. The views in xy-plane with the criterion of (c) non-affine displacements and (d) the atomic shear strain. The views in yz-plane of (e) the non-affine displacements and (f) the atomic shear strain illustration following. Note that the configurational states illustrated in (e) and (f) are in yz-plane to show the surface state.

Supplementary Figure 7: The selected engineering strain for the correlation analysis in the irradiated

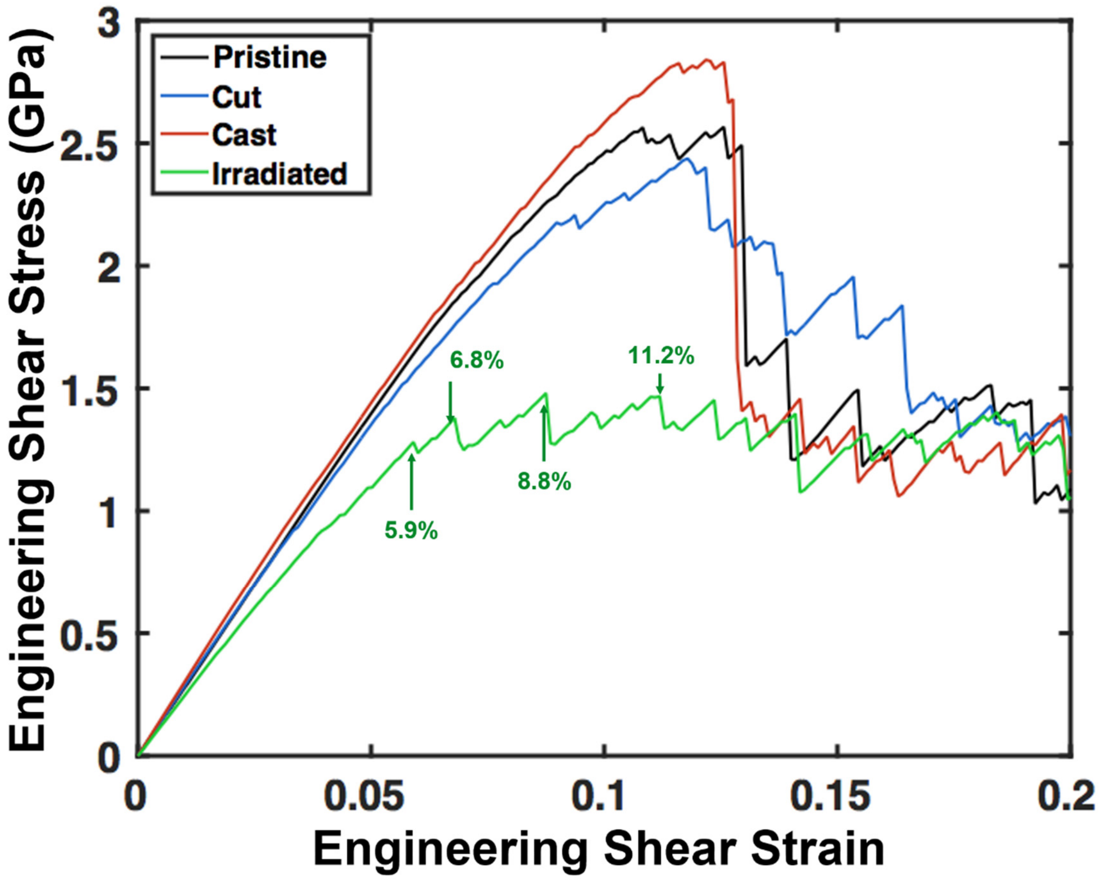

Supplementary Figure S7. 0.2% (Elastic range), 5.9%, 6.8%, 8.8%, 11.2% engineering shear strain steps are selected to analyse the correlation fields of the irradiated.

Supplementary Figure 8: The correlation fields of atomic shear strain and non-affine displacements and corresponding configurational state in the irradiated.

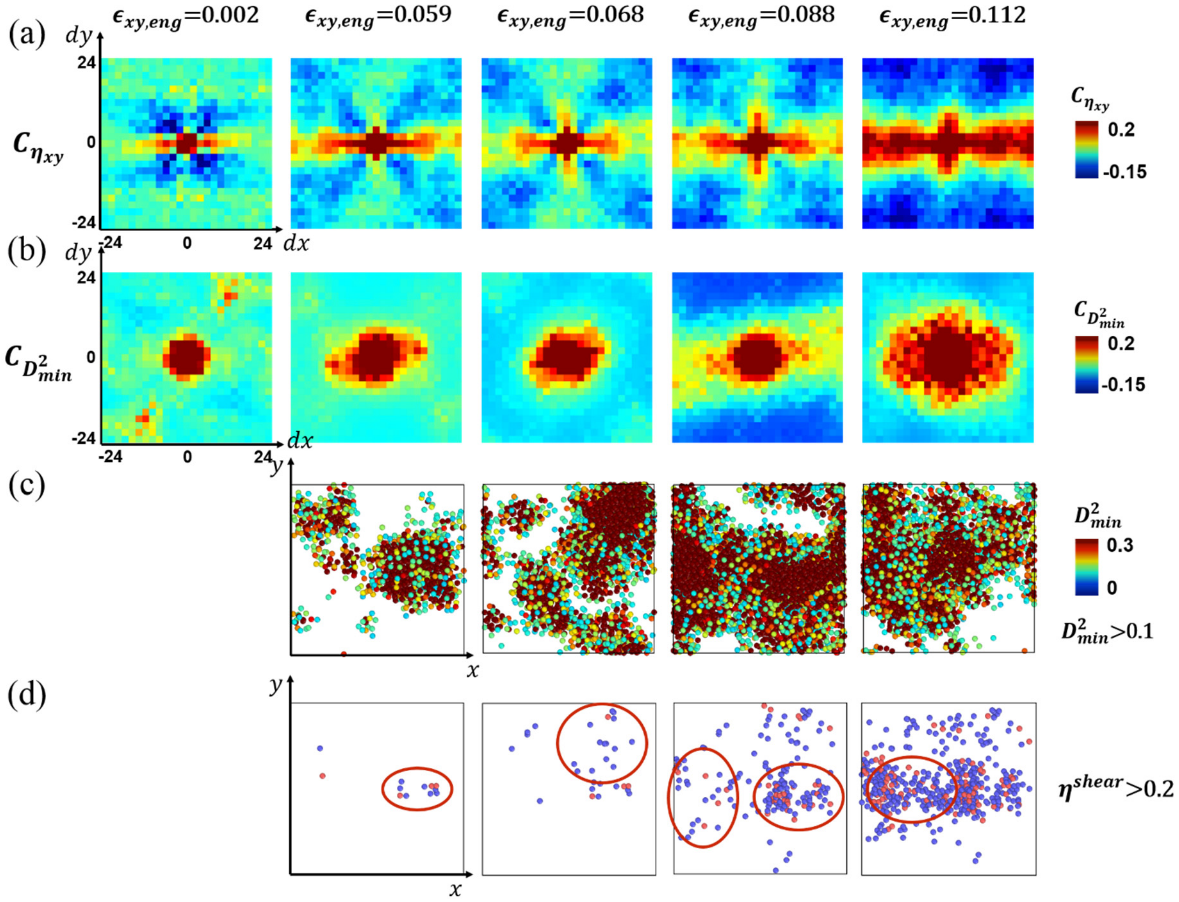

Supplementary Figure S8. The correlation fields of (a) atomic shear strain and (b) non-affine displacements in the irradiated. The views in xy-plane with the criterion of (c) non-affine displacements and (d) the atomic shear strain. Note that the marked red circles represent the activated atoms from the previous engineering strain step.

Supplementary Figure 9: Zr-centered Q_6-participation fraction relationship and the microstructural information and the biaxial stress along the layer

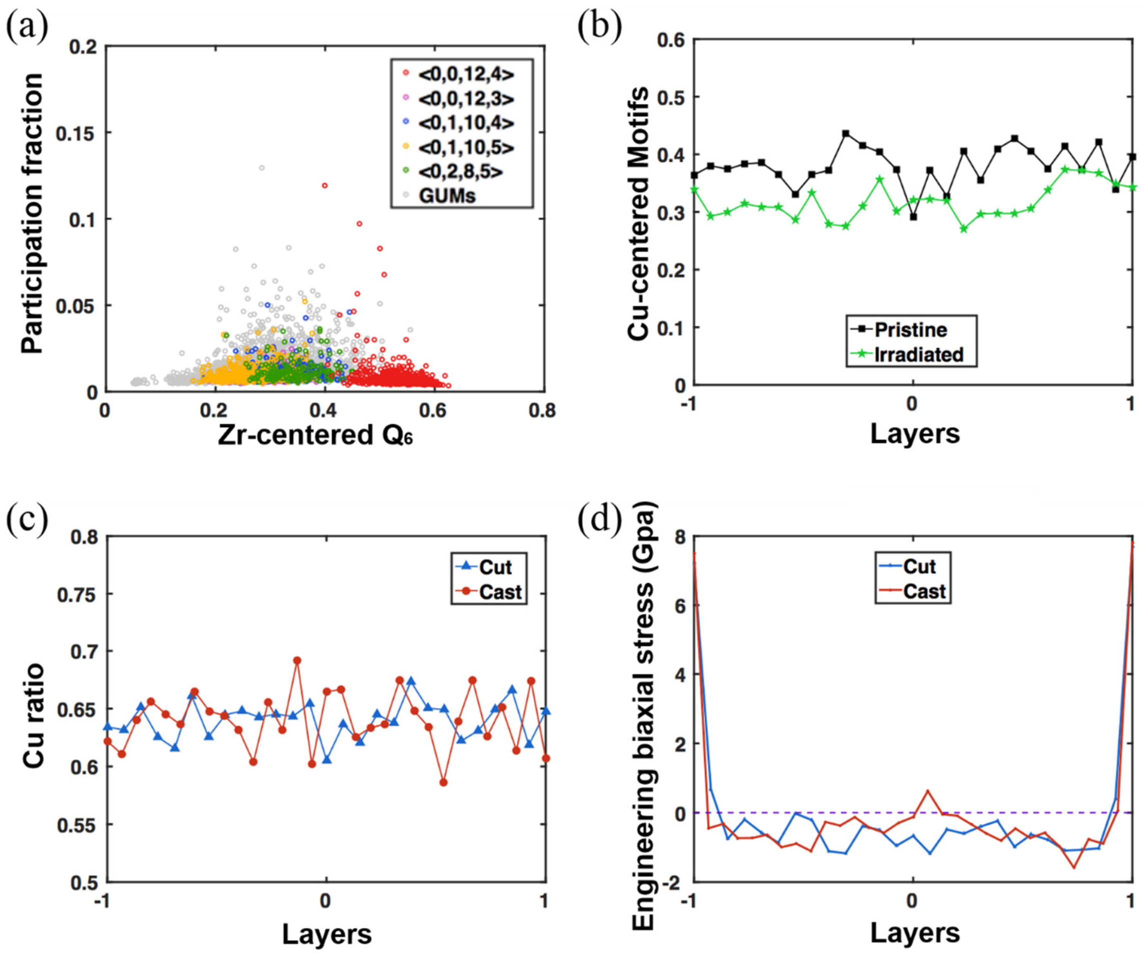

Supplementary Figure S9. (a) Distribution of the participation fraction along the Zr-centered Q_6. Different coloring represents top five most favoured Zr-centered motifs. (b) Ratio of five most favourable Cu-centered motifs along the layer of the pristine and the irradiated bulk. (c) Cu ratio along the layer for the cut and the cast. (d) Averaged biaxial stress in xy plane along the layer for the cut and the cast.

Supplementary Figure 10: 2D cross section of the participation fraction-selected Q_6 relationship

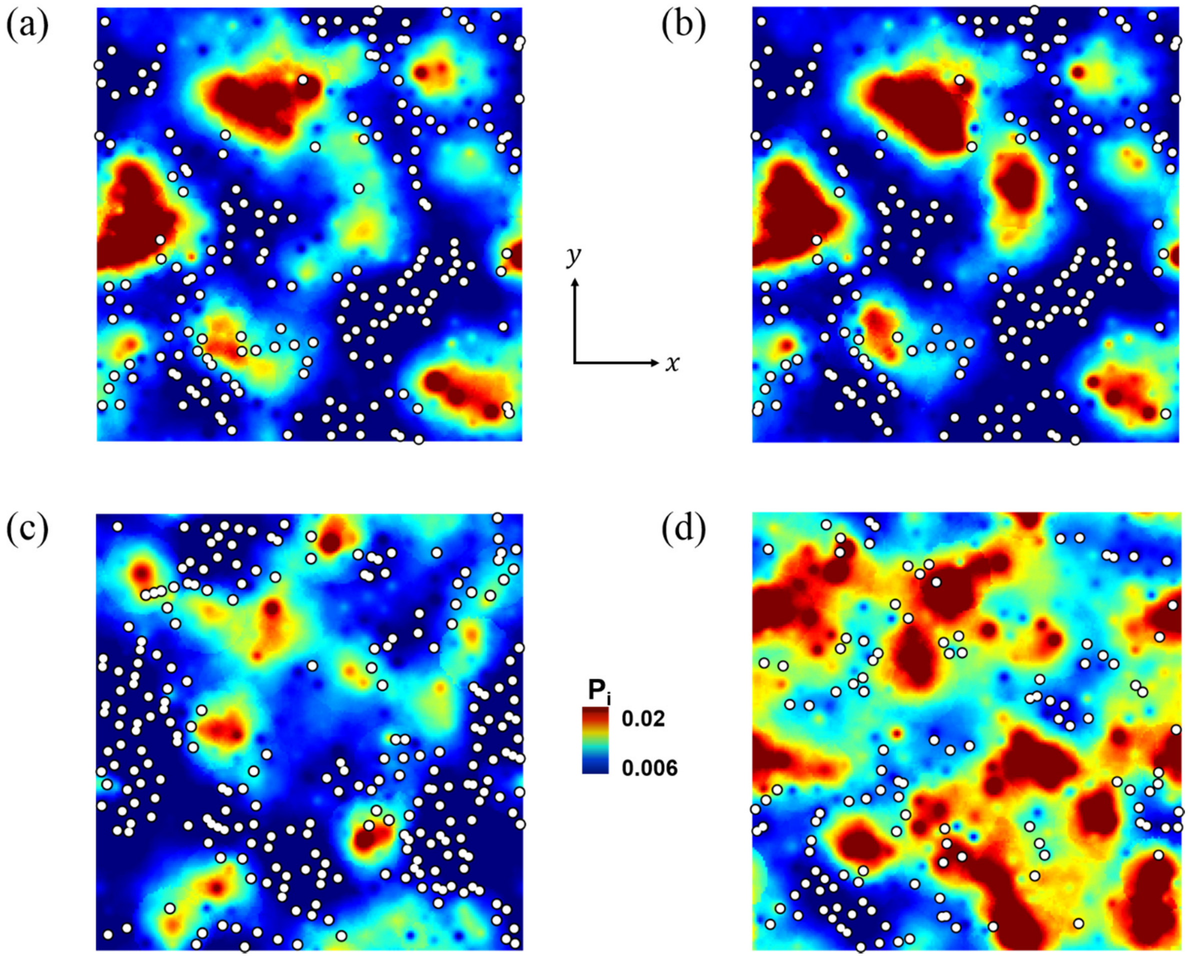

Supplementary Figure S10. Thin slabs in the middle of z-axis (aperiodic axis) with 5 Å are illustrated for (a) the pristine, (b) the cut, (c) the cast, and (d) the irradiated. The background represents the participation fraction and the white circle denotes the Cu atoms in the selected Q_6 range.

Supplementary Figure 11: Structural relaxation process in the cut sample

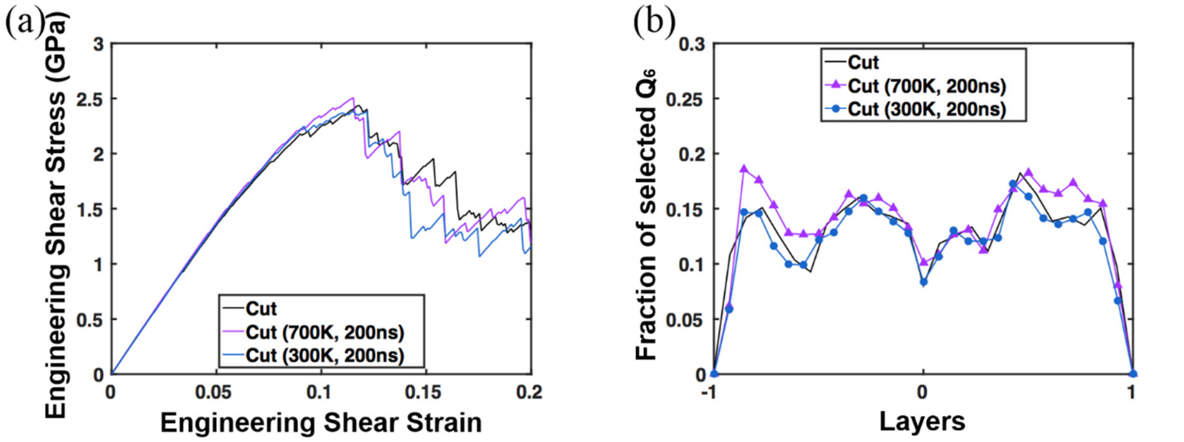

Supplementary Figure S11. (a) Stress-strain curve of the cut sample after 200ns thermal annealing processes at 700K and 300K.black line represents the result of the cut without annealing. (b) Fraction of the selected Cu-centred Q6 parameters along the layer of the cut sample after 200ns thermal annealing processes at 700K and 300K.

Supplementary Figure 12: Distribution of non-affine displacements.

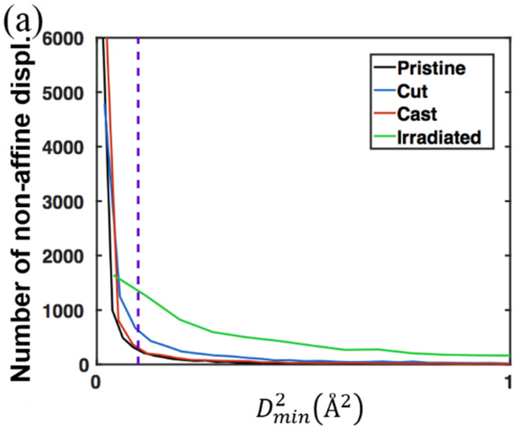

Supplementary Figure S12. (a) Histogram of the number of non-affine displacements of each sample, the pristine, the cut, the cast, and the irradiated. The purple dotted line, 0.1 Å^2, represents the criterion of defining clear irreversible jumps. The non-affine displacements are counted up to 10% engineering shear strain. Note that the strain increment is 0.001 Å.

Supplementary Figure 13: Averaged number of irreversible jumps up to different engineering strain.

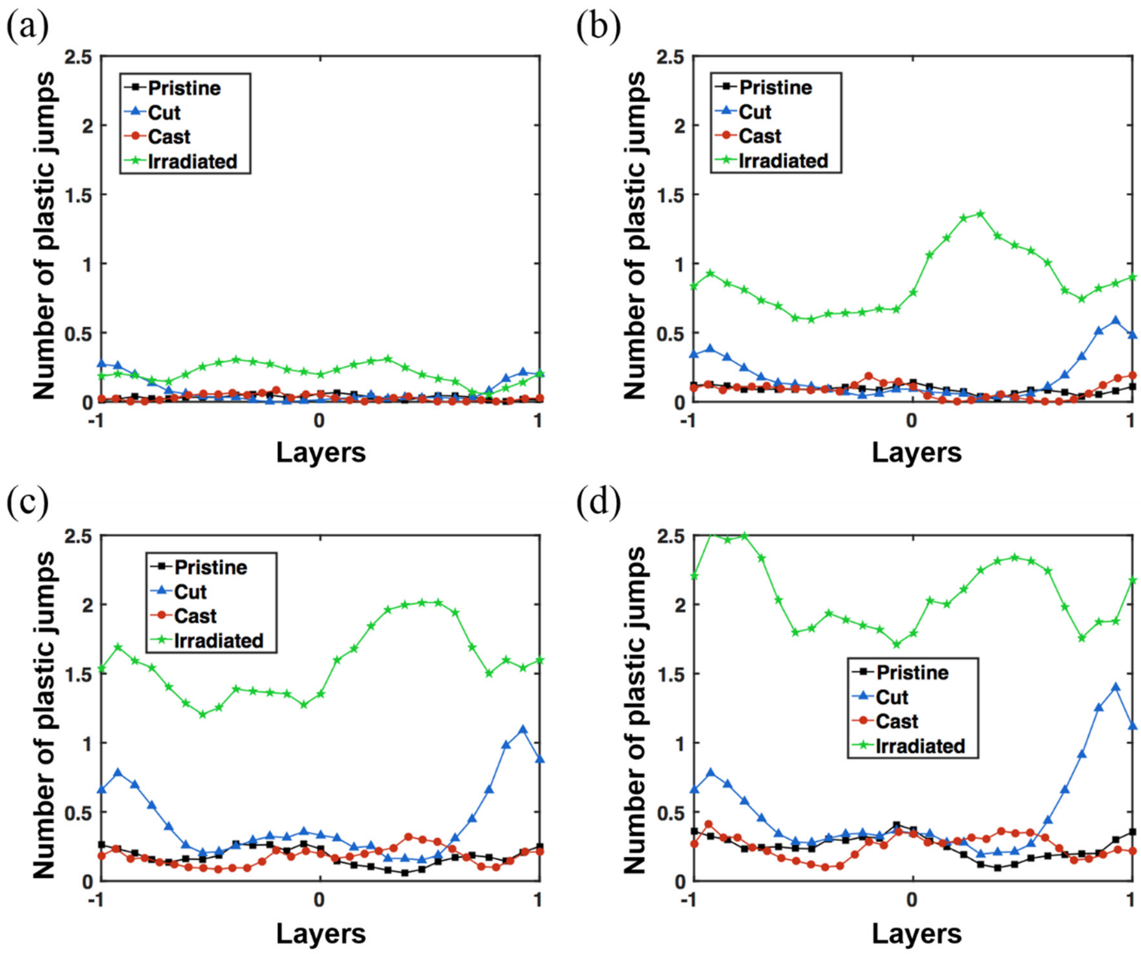

Supplementary Figure S13. Averaged number of irreversible (plastic) jumps along the layers of each sample. The non-affine displacements are counted up to (a) 6% engineering shear strain, (b) 8% engineering shear strain, (c) 10% engineering shear strain, and (d) 11% engineering shear strain.
